# Single pro-adrenomedullin determination in septic shock and 28-day mortality

**DOI:** 10.1186/cc13406

**Published:** 2014-03-17

**Authors:** A Garcia-de la Torre, M De La Torre-Prados, J Parez-Vacas, C Trujillano-Fernandez, A Puerto-Morlan, E Camara Sola, A Garcia-Alcantara

**Affiliations:** 1 University Hospital Puerto Real, Cadiz, Spain; 2 Hospital Virgen de la Victoria, Málaga, Spain

## Introduction

The early phase of septic shock is dominated by severe alterations of the cardiovascular system. The prognostic value of pro- adrenomedullin (pADM), a vasoactive pro-hormone, measured within 24 hours from septic shock onset was assessed.

## Methods

A prospective, observational study in 100 patients >18 years with septic shock in a polyvalent ICU of a university hospital. Demographic, clinical parameters and pADM, C-reactive protein (CRP) and procalcitonin (PCT) were studied during the first 24 hours after admission in 2011. Descriptive and comparative statistical analysis was performed using the statistical software packages StatSoft STATISTICA 7.1 and MedCalc 9.2.1.0

## Results

We analyzed 100 consecutive episodes of septic shock in the ICU. The median age of the study sample was 64 years, interquartile range 16.8 years, 59% were men; the main sources of infection were respiratory tract (48%) and intra-abdominal (24%). The 28-day mortality was 36%. The profile of dead patients showed significantly higher clinical severity scores, APACHE lI (27 vs. 25; *P *< 0.001) and SOFA (12 vs. 10; *P *< 0.001). The area under the curve was 0.72 for pADM, significantly higher than those for CRP (0.62) and PCT (0.65), but similar to those for APACHE II score (0.69) and SOFA score (0.78). Kaplan-Meier survival analysis was significant (*P *= 0.0012) for patients with pADM <1.2 nmol/l. Cox regression analysis also showed statistical significance (*P *= 0.0004) and a likelihood ratio =1.26 per each 1 nmol/l increase in pADM.

## Conclusion

Pro-adrenomedullin is an important prognostic biomarker of survival when measured on admission of septic shock patients to the ICU.

